# Association of LDL-cholesterol subfractions with cardiovascular disorders: a systematic review

**DOI:** 10.1186/s12872-023-03578-0

**Published:** 2023-11-01

**Authors:** Abdolreza Chary, Maryam Tohidi, Mehdi Hedayati

**Affiliations:** 1 Prevention of Metabolic Disorders Research Center, Research Institute for Endocrine Sciences, Shahid Beheshti University of Medical Sciences, PO Box: 19395‑4763, Tehran, Iran; 2 Cellular and Molecular Endocrine Research Center, Research Institute for Endocrine Sciences, Shahid Beheshti University of Medical Sciences, PO Box: 19395‑4763, Tehran, Iran

**Keywords:** Low-density lipoprotein, Cholesterol, LDL subclasses, Small dense LDL, Cardiovascular Disease

## Abstract

**Background:**

Cardiovascular disorders (CVDs) are the leading cause of death worldwide. This study aimed to evaluate the association between low-density lipoprotein (LDL) subfractions and cardiovascular disorders.

**Methods:**

To ensure the rigor of the systematic review, the Preferred Reporting Items for Systematic Reviews and Meta-Analyses (PRISMA) guidelines were used. For this systematic review, a comprehensive search strategy was performed in important databases including PubMed, Scopus, Embase, International Statistical Institute (ISI) Web of Science, and google scholar from 2009 to February 2021. The following terms were used for systematic search: low-density lipoprotein, LDL, subfractions, subclasses, nuclear magnetic resonance, NMR, chromatography, high-pressure liquid, HPLC, cardiovascular disease, cerebrovascular, and peripheral vascular disease. Also, for evaluating the risk of bias, the Newcastle-Ottawa scale was employed.

**Results:**

At the end of the search process, 33 articles were included in this study. The results of most of the evaluated studies revealed that a higher LDL particle number was consistently associated with increased risk for cardiovascular disease, independent of other lipid measurements. Also, small dense LDL was associated with an increased risk of CVDs. There was no association between LDL subfraction and CVDs in a small number of studies.

**Conclusions:**

Overall, it seems that the evaluation of LDL subclasses can be used as a very suitable biomarker for the assessment and diagnosis of cardiovascular diseases. However, further studies are required to identify the mechanisms involved.

**Supplementary Information:**

The online version contains supplementary material available at 10.1186/s12872-023-03578-0.

## Background

Cardiovascular disease is one of the leading causes of mortality in many countries [1]. Various factors, especially increased obesity, an inactive lifestyle, stress, and diseases such as diabetes and dyslipidemia have increased the risk of cardiovascular complications [2]. According to the Adult Treatment Panel III of the Expert Panel of the National Cholesterol Education Program recommendations, increased low-density lipoprotein (LDL) and reduction in high-density lipoprotein (HDL) levels are among the main risk factors for cardiovascular disease [3]. Various results from clinical trials as well as studies evaluating LDL genetic variants have indicated that treatment with statins and other therapies aimed at reducing LDL concentrations can prevent and reduce the risk of cardiovascular events [4–6]. Thus, LDL-lowering therapies are recommended by both European [7] and American guidelines [8] to prevent cardiovascular disease. Also, it has been reported in arteriographic investigations that any interventions to lower serum LDL and elevate HDL concentrations may reduce the rate of arteriographically defined disease progression [9–11].

**Fig. 1:**
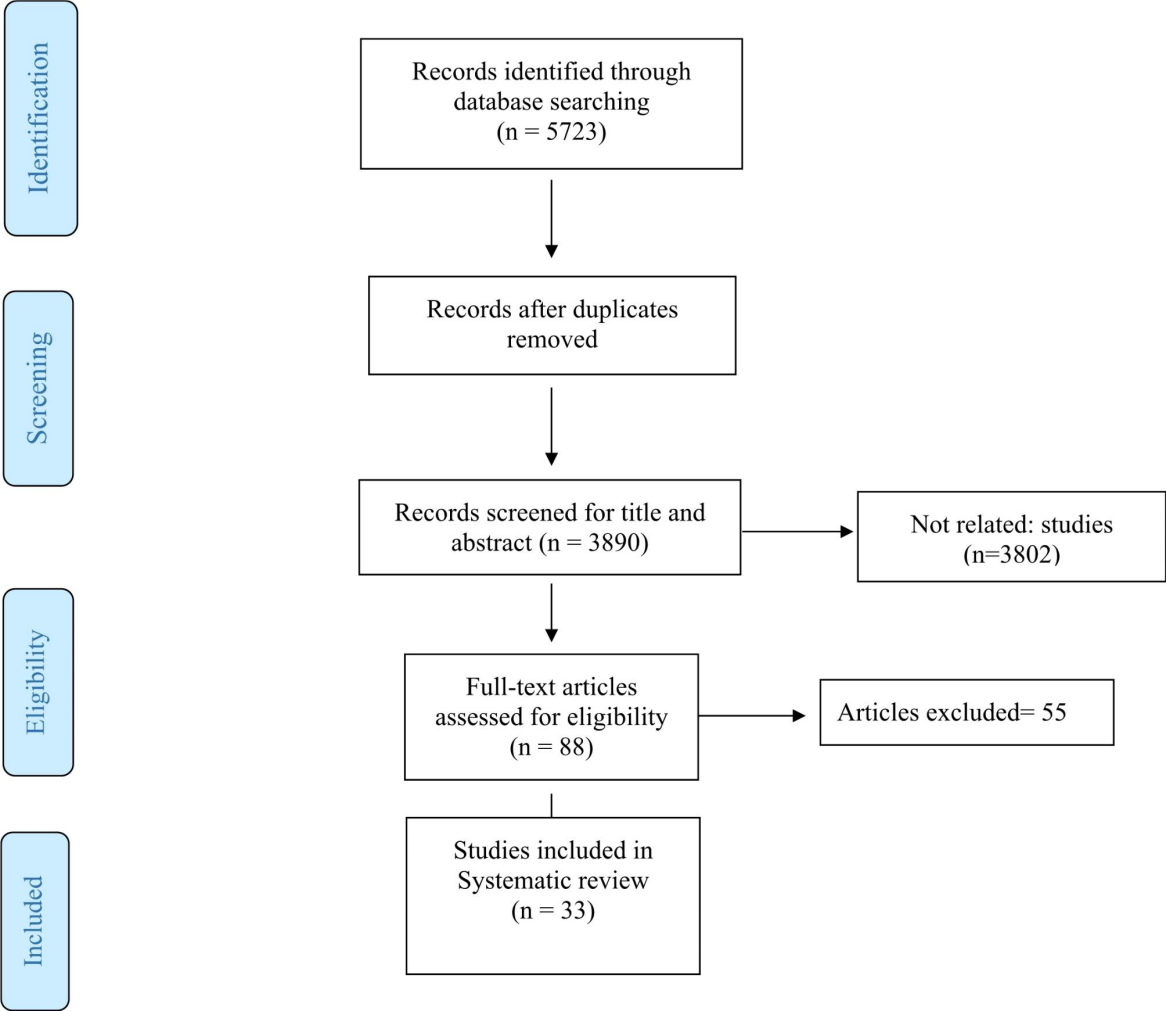
Flow chart of study selection

Recognizing the role of LDL as a serious risk factor for cardiovascular disease, one of the questions that has arisen is whether the size of LDL particles and different subtypes of LDL plays the same role in the etiology of cardiovascular failure. It has been reported that some conditions such as metabolic syndrome, diabetes, familial combined hyperlipidemia, and hyperapobetalipoproteinemia (hyper-apoB) would elevate the concentration of small atherogenic LDL and lead to cardiovascular disease, as summarized in Fig. 1 [12, 13].

On the other hand, it has been shown that the effect of drug treatment such as statin therapy or Proprotein Convertase Subtilisin/Kexin type 9 (PCSK9) inhibitors was not the same in patients with high LDL levels, raising suspicion among researchers and physicians that differences in observed effects may be due to different concentrations of LDL subclasses [14–16]. Different terms are used to describe the characteristics and distribution of LDL particles such as LDL subclasses, subfraction and particle concentration, though all of these terms have almost the same meaning, and more attention has been paid to LDL subclasses since subclass separation techniques. Some studies have reported that small dense LDL (sdLDL) particles are at greater atherogenic risk than larger, less dense LDL, while some results are contradictory [17, 18]. Indeed, some studies have suggested that sdLDL are more taken up by macrophages and are at higher risk for oxidation. On the other hand, these particles easily penetrate into the subendothelial space and attach to the arterial wall, thus increasing the risk of atherosclerosis [19, 20]. A systematic review study conducted in 2009 by Stanley et al. [21] assessed the association between LDL subgroups and the incidence of cardiovascular Outcomes. However, the results of this study were contradictory and the authors of this article recommended that further studies be conducted to identify the mechanisms involved.

Due to the contradictory results and limitations mentioned in different studies, this updated systematic review study aimed to investigate the relationship between different LDL subclasses and the risk of cardiovascular disease.

## Method

### Search strategy

To ensure the rigor of this systemic review, the Preferred Reporting Items for Systematic Reviews and Meta-Analyses (PRISMA) guidelines were used [22]. For this systematic review, a comprehensive search strategy was employed in important databases including PubMed, Scopus, Embase, International Statistical Institute (ISI) Web of Science, and google scholar from 2009 to February 2021. In order not to miss an article, manual searching was performed. For systematic search, the following search strategy was used: (“Low-density lipoprotein” OR Cholesterol, LDL OR LDL OR lipoprotein) AND (particle size.mp. OR subfractions OR subclasses OR “Nuclear Magnetic Resonance” OR Biomolecular/ OR exp Magnetic Resonance Spectroscopy/ OR “nuclear magnetic resonance” OR NMR OR “magnetic resonance spectroscopy” OR Chromatography OR “High-Pressure Liquid” OR HPLC OR ultracentrifugation.mp OR centrifugation.mp OR Electrophoresis) AND (“cardiovascular disease” OR cerebrovascular OR peripheral vascular disease OR Cardiovascular OR Cardio* OR Atherosclerosis).

### Criteria for selecting articles

Table 1 summarizes the population, interventions, comparators, and outcomes (PICOS) criteria for eligibility of studies. Articles were included in this study if they met the following criteria: prospective, longitudinal, and cross-sectional design which evaluated the association between LDL subfractions and cardiovascular disease, participation of at least 10 people in the study, serum (or plasma) samples must have been obtained before determination of outcomes, evaluation of specific clinical outcome such as minimum lumen diameter.

The results of a systematic search for initial screening were entered into the EndNote software. After eliminating duplicate studies, the two authors independently evaluated the titles and abstracts of the articles. In the second stage, the researchers evaluated the full text of the remaining articles and the studies that met the necessary criteria were included in the final analysis. Also, studies with low methodological quality were detected. Data such as authors, country of study, method of assessing LDL levels, cardiovascular disease, etc. were extracted.

### Risk of bias assessment

For evaluating the risk of bias, the Newcastle-Ottawa scale was used [23]. NOS was developed to evaluate the quality of nonrandomized studies, including cross-sectional, case-control, and cohort studies. This assessment allowed a total score of up to 9 points. The NOS for cohort studies was divided into three groups: selection of cohort (4 points), comparability of cohort (2 points), and assessment of outcome (3 points). The quality of the study was considered high or moderate if the sum score was ≥ 8 points or between 5 and 7 points, respectively.

**Table 1:** PICOS criteria for inclusion and exclusion of studies

Parameter	
**Population**	Participants with abnormal levels of LDL subfraction
**Exposure (or Intervention)**	For studies that examined the association between serum LDL subfraction concentration and the risk of cardiovascular diseases, exposure is " LDL subfraction”
**Comparators**	Subjects with normal levels of LDL subfraction
**Outcome**	Risk of cardiovascular diseases

## Results

### Characteristics of the included studies

In total, after searching the mentioned databases, 5723 studies entered the Endnote 20 software and after removing duplicate articles, 3890 articles remained for initial screening. Following the initial screening, 88 studies were chosen to evaluate the full text of these articles, and among them, finally, 33 articles were included in this study (Table 2). The flow chart of study selection has been presented in Fig. 1.

**Table 2:** Characteristics of the included studies

First Author	Type of study	Subjects	LDL subclass type	Evaluation Method	Main results	Total quality score
Pallarés (2021)	Cross-Sectional	Adults	LDL-Ps, L-LDL-P, M-LDL-P, S-LDL-P	NMR spectroscopy	higher CVDs rate and systolic blood pressure were significantly associated with abnormalities in the number of S-LDL-P	8
Antonio (2021)	Case-Control	women without CVD	LDL-P	NMR spectroscopy	LDL-related variables were the most strongly associated with atherosclerosis	9
Duan (2020)	Cross-Sectional	hospitalized patients with Acute ischemic stroke	LDL-1, LDL-2, LDL-3, LDL-4, LDL-5 to 7	polyacrylamide gel electrophoresis technique	LDL-3 and LDL-4 levels, were significantly positively correlated with AIS	9
Rodríguez (2019)	Cross-Sectional	middle- aged US- White and Japanese men	Small LDL- P, Large LDL- P,	NMR spectroscopy	total LDL- P and small LDL- P were significantly associated with coronary heart disease	8
Kidawa (2019)	Cross-Sectional	patients with Acute Coronary Syndromes (ACS)	LDL 1-LDL 5, IDLA	NMR spectroscopy	Patients with multi-vessel CADs disease had higher levels of LDL3 subfraction and IDL-C and a lower proportion of IDLA	7
Notarnicola (2018)	Prospective Cohort	cardiovascular diseases	Small LDL-C, Large LDL-C	NMR spectroscopy	Higher small LDL concentration was associated with higher CVDs mortality	7
Llauradó (2019)	Case-Control	participants with T1DM	Small LDL-C, Large LDL-C	NMR spectroscopy	Higher small LDL concentration was associated CVDs risk factors.	8
Chang (2019)	Cross-Sectional	Rheumatoid Arthritis Patients	LDL 1-LDL 5	fast-protein liquid chromatography	Plasma L5 levels were significantly higher in patients with subclinical atherosclerosis	9
Aneni (2019)	Cross-Sectional	High-Risk Individuals	LDL-VS, LDL-S, LDL-M, LDL-La	gas-phase differential electrical mobility	Higher concentrations of large LDL were seen among those with no coronary artery calcification. small and medium LDL particles were seen among those with coronary artery calcification	9
Žitňanová (2019)	Case-Control	patients with acute ischemic stroke	LDL 1-LDL 5	electrophoresis	sdLDL was significantly higher in patients after acute ischemic stroke	7
Schulte (2018)	Cross-Sectional	Patients with Chronic inflammatory diseases	lbLDL-C, sdLDL-C	gas-phase differential electrical mobility	The sdLDL/LDL ratio was higher in patients with cardiovascular risk factors.	8
Chu (2018)	Cross-Sectional	patients with coronary artery disease	L1-L5	liquid chromatography	Plasma L5 levels were significantly higher in patients with coronary artery disease	6
Aday (2018)	Prospective Cohort	women ≥ 45 years old free of cardiovascular disease	Small LDL-C, Large LDL-C	NMR spectroscopy	sdLDL-C particle concentration, but not LDL-C, were associated with peripheral artery disease (PAD)	7
Siarnik (2017)	Cross-Sectional	Patients with acute ischemic stroke	IDL1, IDL2,IDL3, LDL1, LDL2, LDL3-7	Lipoprint LDL System	LDL1 was significantly associated with acute ischemic stroke	8
Shiffman (2017)	Case-Control	Adult participants	small LDL subfraction (LDL-VS), large LDL subfraction	ion mobility	LDL-VS was associated with CVDs	6
Pokharel et al. (2017)	Cross-Sectional	Patients with Myocardial Infarction	pattern A consisted of a preponderance of large, buoyant LDL subclass, while pattern B consisted mainly of small, dense LDL subclass	ultracentrifugation	when LDL pattern B was compared with LDL pattern A, there was significant 60% relative reduction in CV mortality.	9
Lawler et al. (2017)	Cross-Sectional	Individuals With Low Low-Density LipoproteinCholesterol	total LDL-p, small and large LDL-p, intermediate density lipoprotein [IDL-p]	NMR spectroscopy	Smaller LDL size was a marker of increased risk, but this was no longer significant after additionally adjusting for LDL-p	9
Gluba‑Brzózka (2017)	Case-Control	end‑stage renal disease (ESRD) patients	LDL1-LDL7	Lipoprint LDL System	There wasn’t significant association between LDL subclasses and cardiovascular abnormalities.	8
Shen et al. (2016)	Case-Control	ischemic stroke patients	L5	NMR spectroscopy	levels of plasma L5 were significantly higher in acute ischemic stroke patients than in controls	6
Steffen et al. (2015)	Cross-Sectional	Adult participants	total LDL particles (LDL-P)	NMR spectroscopy	There was a significant association between LDL-P and CHD events	7
Vishnu et al. (2014)	Cross-Sectional	middle-aged men	Large LDL-P, Small LDL-P, Total HDL-P	NMR spectroscopy	arterial stiffness had a significant positive association with small LDL-P and significantly inversely associated with large LDL-P and LDL size.	8
Nishikura (2014)	Cross-Sectional	Patients with Coronary Artery Disease	sdLDL-C, Large LDL-C	gradient gel electrophoresis	Those who experienced cardio-vascular events had higher levels of sdLDL-C, sdLDL-C/LDL-C, and LDL-C/high-density lipoprotein cholesterol (HDL-C) ratios	7
Jug et al. (2014)	Cross-Sectional	Patient at Intermediate Cardiovascular Risk	Small LDL-C, Large LDL-C	NMR spectroscopy	LDL pattern B (predominance of small dense particles) emerged as an independent predictor of coronary calcium	6
Gerber et al. (2013)	Prospective Cohort	Patients with (pre)diabete	sdLDL-C	gradient gel electrophoresis	Higher concentration of sdLDL-C was associated with intima media thickness	8
Cure et al. (2013)	Case-Control	Patients with ischemic stroke	Small LDL-C, Large LDL-C	NMR spectroscopy	The mean LDL particle size was smaller in patients with stroke than in the controls	7
Okumura et al. (2013)	Cross-Sectional	Patients with Endothelial Dysfunction	sdLDL-C	HPLC	small LDL cholesterol emerged as an independent determinant of Endothelial Dysfunction among lipoprotein subfractions	8
Lakshmy et al. (2012)	Cross-Sectional	young Indian industrial population	sdLDL-C	polyacrylamide gel electrophoresis technique	small dense LDL was associated with cardiovascular risk factors	6
Hirayama et al. (2012)	Cross-Sectional	adult	Small LDL- C	NMR spectroscopy	Small LDL- C is a emerging risk factor for cardiovascular disorder	5
Prado et al. (2012)	Cross-Sectional	asymptomatic adults at intermediate risk of cardiovascular disease	Large-pattern LDL(Pattern A) was defined as 23.0–20.6 nm, and small-pattern LDL (Pattern B) was defined as 20.5–18.0 nm.	NMR spectroscopy	small-pattern LDL (Pattern B) was an independent predictor of coronary artery calcification	7
Zeljkovic et al. (2012)	Cross-Sectional	Patients with acute ischemic stroke	LDL I-IV	gradient gel electrophoresis	AIS patients had significantly more LDL III and IVb, but less LDL I and II particles.	8
Chung et al. (2010)	Case-Control	Patients with rheumatoid arthritis	Small LDL-C, Large LDL-C	NMR spectroscopy	There wasn’t any significant association between small LDL level with coronary artery calcification (CAC)	7
Rizzo et al. (2009)	Prospective Cohort	subjects with the metabolic syndrome	sdLDL-C, Large LDL-C	gradient gel electrophoresis	small, dense LDL was a predictor of CVDs	8
Mora et al. (2009)	Prospective Cohort	Healthy women	Small LDL-C, Large LDL-C	NMR spectroscopy	CVDs risk prediction associated with LDL subclass profiles evaluated by NMR	9

From those articles included, based on the design of studies, 20 studies had cross-sectional, 5 prospective cohort design, and 8 studies had a case-control design.

For evaluating LDL subclasses, 17 studies had employed NMR spectroscopy, 8 studies gel electrophoresis, one study HPLC, two studies liquid chromatography, one study electrical mobility, and four studies the ion mobility ultracentrifugation and lipoprint system.

Based on the Newcastle–Ottawa checklist, regarding the score of methodological quality, all included studies except six [4, 24–28] had high quality (more than 7 scores) (Table 3).

**Table 3:** Quality assessment of included studies

First author	Selection	Comparability	Outcome	Total
Pallarés	4	1	3	8
Antonio	4	2	3	9
Duan	4	2	3	9
Rodríguez	3	2	3	8
Kidawa	3	1	3	7
Notarnicola	4	1	2	7
Llauradó	4	2	2	8
Chang	4	2	3	9
Aneni	4	2	3	9
Žitňanová	3	2	2	7
Schulte	3	2	3	8
Chu	3	1	2	6
Aday	4	2	2	8
Siarnik	3	2	2	7
Shiffman	3	2	1	6
Pokharel	4	2	3	9
Lawler	4	2	3	9
Gluba‑Brzózka	4	2	2	8
Shen	3	2	1	6
Steffen	4	2	1	7
Vishnu	4	2	2	8
Nishikura	4	2	1	7
Jug	3	1	2	6
Gerber	4	2	2	8
Cure	3	2	2	7
Okumura	4	2	2	8
Lakshmy	3	2	1	6
Hirayama	3	1	1	5
Prado	3	2	2	7
Zeljkovic	4	2	2	8
Chung	4	2	1	7
Rizzo	4	2	2	8
Mora	4	2	3	9

### NMR-measured LDL subfractions

Among the studied studies, 17 studies had utilized the NMR method to evaluate LDL subfraction. Pallarés et al. [29] in a cross-sectional study conducted on 400 participants, reported that subjects with higher concentrations of small LDL particle size had a higher chance of developing cardiovascular disease. Also, Notarnicola et al. [30] showed that higher concentrations of sdLDL would increase the risk of mortality in patients with CVDs. In line with the two studies mentioned, other studies reported similar results [24, 25, 31–34]. Further, some studies have specifically examined the association between LDL subtypes and the incidence of various cardiovascular diseases. Cure et al. in a case-control study among patients with ischemic stroke reported that the level of sd‑LDL was 8.2 ± 7.8 mg/dL in the stroke group, which was significantly higher than the control group. However, the concentration of total LDL and large particles of LDL did not differ significantly between the two groups [35]. Also, Zeljkovic et al. in a cross-sectional study evaluated the concentration of various LDL subfractions among 100 patients with acute ischemic stroke and found that acute ischemic stroke (AIS) patients had significantly more LDL III and IV, but fewer LDL I and II particles [36].

### LDL subfractions and coronary artery calcification (CAC)

Aneni et al. in a study conducted among 182 high cardiometabolic risk participants evaluated the association between LDL subfraction and risk of CAC. The result of this study revealed that subjects with higher concentrations of small/medium LDL subfractions had a higher risk for CAC odds compared to the participants with large LDL subfractions [37]. Prado et al. in a cross-sectional study evaluated the association between LDL subfractions and CAC among the 284 adults at intermediate risk of cardiovascular disease. They reported that the risk of CAC was 3.7 times higher in people with higher tertile of LDL particle (LDL-P) number [38]. Also, Jug et al. showed that serum concentration of small dense LDL lipoprotein was an independent predictor of CAC among the 410 patients at intermediate cardiovascular risk [26]. However, contrary to the results of the three studies mentioned, Chung et al. in a case-control study among 139 patients with rheumatoid arthritis concluded that there was no significant association between small LDL concentration and CAC [39].

### LDL subfractions and stroke risk as well as atherosclerosis

Antonio et al. in a case-control study among 112 women with type 1 diabetes reported that patients with higher LDL particle (LDL-P) had a higher risk for atherosclerosis. Also, participants with higher concentrations of small LDL showed a higher risk for atherosclerosis and stroke [40]. Further, Duan et al. in a study on 566 patients with AIS reported that patients with AIS had a significantly higher concentration of LDL-3, LDL-4, and LDL-5 subclasses as well as lower concentration of LDL1 compared to the non-AIS participants [41]. A similar finding was observed in Chang et al. study [42]. In addition, Žitňanová et al. in a cross-sectional study which evaluated the association between LDL subfraction and AIS outcome, found that the serum concentration of anti-atherogenic large LDL1 subfractions was significantly lower in patients with AIS, and in contrast, they have a higher concentration of LDL3 and LDL5, which atherogenic properties [43].

## Discussion

The present study has been a systematic review evaluating the association between LDL subclasses and cardiovascular disease. The results of the study revealed that participants with higher concentrations of small dense LDL were at a higher risk for CVDs. Additionally, we found that subjects with CVDs or those at risk for CVDs had higher concentrations of atherogenic LDL subclasses, such as LDL3 and LDL5.

In numerous countries, cardiovascular disease has emerged as a primary cause for mortality. Several articles have put different etiologies and theories for CVDs, among which a significant one is the rise in LDL levels, comprising seven subclasses (LDL-1 to LDL-7) [44]. The studies conducted so far on how LDL subclasses are linked to the development of different cardiovascular diseases have produced inconsistent findings [45, 46]. As the importance of evaluating LDL subclasses to predict cardiovascular disease has expanded, various methods have been developed to evaluate them [47]. For evaluating LDL subclasses, 17 studies had used NMR spectroscopy, 8 studies gel electrophoresis, one study HPLC, two studies liquid chromatography, one study electrical mobility, and four studies the ion mobility ultracentrifugation and lipoprint system. Most of the studies included in this systematic review had utilized the NMR spectroscopy method. NMR evaluates the number of LDL particles by applying a particular formula to measure the area and identifying the signal from the combined quantity of terminal methyl groups of the lipid present within the particle. Nonetheless, certain studies have employed the ultracentrifugation technique to assess LDL subgroups, where the separation of LDL subgroups is based on their density [48].

Although dyslipidemia is recognized as a traditional risk factor for cardiovascular disease, many patients with a history of acute vascular events have normal LDL levels. Concurrently,

Some people without any clinical or laboratory signs of CVDs exhibited higher concentrations of LDL-cholesterol [49, 50]. These findings promoted researchers to investigate and identify atherogenic and non-atherogenic subclasses of lipoproteins profiles. Some of the studies revealed that an atherogenic profile, characterized by elevated concentrations of VLDL, IDL1–3, small HDL, and especially by high levels of small dense LDL (LDL3–7) subfractions, can increase the risk of atherogenesis and CVDs. Meanwhile, the anti-atherogenic profile of lipoprotein subclasses, which includes a higher level of subtypes such as LDL1-2, large HDL, intermediate HDL and by only trace concentrations of LDL3–7 subfractions, has been identified by improving the body’s defenses against cardiovascular disease [51–53].

Despite numerous clinical and in vitro investigations, the precise mechanism behind the atherogenic effects of certain LDL subclasses remains uncertain. One proposed mechanism is that small, high-density LDL particles have a weaker binding affinity to hepatic LDL receptors, thus prolonging their clearance process [54]. Other researchers have suggested that elevated concentrations of small, dense LDL particles would increase their binding to intimal proteoglycans [55]. Additionally, LDL subclasses with smaller particles and higher density are more susceptible to oxidation, which leads to the formation of macrophage-derived foam cells, the hallmark of atherosclerotic plaques [56]. Furthermore, some studies have linked specific LDL subclasses to endothelial dysfunction [57].

The significance of examining lipoprotein subclasses has become so great that some associations that deal with cardiovascular diseases have included this field in their recommendations. While the American Heart Association still issues guidelines for treating CVD patients based on LDL levels, certain European associations, such as the European Society of Cardiology, suggest recommendations based on the level of LDL subclasses, such as sdLDL [58]. Evaluation of LDL subclasses can be used as a useful biomarker to identify people at risk for cardiovascular disease and to provide early preventive recommendations including diet and exercise [59].

The present review has been an updated systematic review, which evaluated observational studies evaluating the association between LDL subclasses and CVDs risk factors, incidence, and outcome. It has assessed results from 33 studies with a total of 12,320 subjects, providing substantial statistical power. Also, most of the studies had employed the same method to measure LDL subclasses, which is a standard method, though the evaluation method was different in some studies.

According to our knowledge, the present study has been the first systematic study examining the relationship between LDL subclasses and cardiovascular diseases. The current study had many strengths, including the systematic and comprehensive search across various databases, accurate and complete screening process, evaluation of methodological quality of studies with standard tools, and comprehensive review of all subclasses of LDL. Several limitations warrant discussion. One of the most important limitations of this study was the impossibility of meta-analysis due to the high heterogeneity of the studied outcomes. Also, the participants included in the studies in terms of health status or type of disease was a wide range, which can affect the accuracy of the results. Also, the consequences evaluated in different studies were adjusted for different confounding variables, which in turn can affect the accuracy of the results.

## Conclusions

In conclusion, the assessment of LDL subclasses can be a useful biomarker for the evaluation and diagnosis of cardiovascular diseases. The results of this systematic review suggest that higher concentrations of small dense LDL particles are associated with increased risk for cardiovascular disease. However, further studies are needed to identify the mechanisms involved and to determine the clinical utility of measuring LDL subfractions in the management of cardiovascular disease.

### Electronic supplementary material

Below is the link to the electronic supplementary material.

Supplementary Material 1

## Data Availability

The datasets used and/or analyzed during the current study are available from the corresponding author on reasonable request.

## Abbreviations

CAC: Coronary artery calcification
CVDs: Cardiovascular disorders
LDL: Low-Density Lipoprotein
HDL: High-Density Lipoprotein
PICOS: Population,interventions,comparators and outcomes
PRISMA: Preferred Reporting Items for Systematic Reviews and Meta-Analyses
ISI: International Statistical Institute
AIS: Acute Ischemic Stroke
HPLC: High-performance liquid chromatography
NMR: Nuclear magnetic resonance spectroscopy
NOS: Newcastle-Ottawa scale
sdLDL: Small dense LDL
PCSK9: Proprotein Convertase Subtilisin/Kexin type 9
