# Supplementary material for: Association of LDL-cholesterol subfractions with cardiovascular disorders: a systematic review
Source: BMC Cardiovasc Disord. 2023 Nov 1;23:533. doi: 10.1186/s12872-023-03578-0 (PMC10621218; doi:10.1186/s12872-023-03578-0)
Supplement: Supplementary file 1 — Supplementary Material 1 [file 12872_2023_3578_MOESM1_ESM.docx]

Dear Reviewer,

Thank you for taking the time to review our article titled " Association of LDL-cholesterol subfractions with cardiovascular disorders: A systematic review." We appreciate your thoughtful comments and suggestions, which have helped us to improve the quality of our manuscript.

We have carefully considered each of your comments and have made the following revisions to the manuscript:

Reviewer 1
Abdolreza et al. aimed to evaluate the association between Low-Density Lipoprotein (LDL) subfractions and cardiovascular disorders. Their results shown that the higher LDL particle number was consistently associated with increased risk for cardiovascular disease, independent of other lipid measurements. This is interesting because small dense LDL indeed plays a major role in CVDs progression.

Response: Thank you very much for your valuable comment.

Reviewer 2
I reviewed the manuscript entitled “Association of LDL-cholesterol subtractions with cardiovascular disorders: A systematic review”. The manuscript is a well-written article, in the scope of the journal, but certain shortcomings should be addressed before its publication:

Thorough language and grammar editing is required. Many of the phrases are hard to understand, making the whole manuscript hard to follow.

The terms used for the review and the inclusion and exclusion criteria should be added to the abstract.

Response: Dear reviewer, thank you for your valuable comments, it has been added to the manuscript in line number of 36-39.

What does LDL particle number mean?

Response: Dear reviewer, LDL particle number (LDL-P) measures the actual number of LDL cholesterol particles in your blood. Patients with small, dense LDL particles have also been shown to have a 3-fold increased risk of myocardial infarction, independent of age, sex, and relative weight. Small LDL particle size has also been suggested to be an additional risk factor for coronary artery disease.

• Please elaborate on reason for choosing Newcastle-Ottawa Scale for quality assessment.

Response: Dear reviewer, NOS was developed to evaluate the quality of nonrandomized studies, including cross sectional, case-control and cohort studies.

The Newcastle-Ottawa Scale (NOS) is an ongoing collaboration between the Universities of Newcastle, Australia and Ottawa, Canada. It was developed to assess the quality of nonrandomised studies with its design, content and ease of use directed to the task of incorporating the quality assessments in the interpretation of meta-analytic results. A 'star system' has been developed in which a study is judged on three broad perspectives: the selection of the study groups; the comparability of the groups; and the ascertainment of either the exposure or outcome of interest for case-control or cohort studies respectively. The goal of this project is to develop an instrument providing an easy and convenient tool for quality assessment of nonrandomised studies to be used in a systematic review.

The face/content validity of the NOS has been established based on a critical review of the items by several experts in the field who evaluated its clarity and completeness for the specific task of assessing the quality of studies to be used in a meta-analysis. Also, the NOS has been refined based on experience using it in several projects, in particular, a project assessing the association of CHD with hormone replacement therapy in postmenopausal women and a project assessing the association of connective tissue disease with silicone breast implants.

In the results, you mentioned that you found only 88 of the 3890 articles. This is a very low number, what were the approaches you made to find the full texts?

Response: Dear reviewer, I think you misunderstood what we mean. In the first stage of screening of systematic review studies, as you know, there is no need to check the full text of the articles, and in this stage, by checking the title of the articles and their abstract, the articles that were completely unrelated to our topic were removed. After this stage, the articles that enter the second phase (which was 88 studies in our study) were reviewed for a more detailed review of their full text, considering that they may be related to our work based on the title and abstract. So, in general, these 88 articles were evaluated for further investigation in the second phase of screening. We had the ability to access the full text of all 3890 articles, but in the first stage of screening, as mentioned, according to the standards of the Cochrane Institute, there is no need to review the full text, and the full text of the articles is reviewed in the second phase after removing irrelevant articles.

• The discussion part is hard to follow and therefore should be reorganized and rewritten.

Dear reviewer, we have tried to rewrite many parts of the discussion section to improve it according to your valuable comments.

In the end, it is still not clear what is the conclusion of the authors, how this finding is going to help clinical practice and what is the recommendations for future studies.

Response: Dear reviewer, it has been corrected according your valuable comments.

Please provide a citation for Figure 1.

Response; Dear reviewer, Figure number one shows the flowchart of the screening process and the process of selecting articles for the present study. For this reason, it has not been inferred from any other study that needs citation. In fact, this figure only shows the screening process of our study. For this reason, similar to all the other systematic studies, in no study they cite Figure 1, which is a screening flowchart.

The homogeneity of the results should be included and briefly described in the result section of the article.

Response; Dear reviewer, Because the studies included in this study were completely heterogeneous in terms of the investigated outcomes, it was not possible to perform a meta-analysis for the present study and it was only done in a systematic way. In fact, we could report the degree of homogeneity and heterogeneity when the I^2^ index was calculated for the investigated factors, which was only possible in meta-analysis.

I advise you to add the strengths of your study in the discussion section.

Response: Dear reviewer, it has been added to the discussion section based on your valuable comment.
